# First clinical implementation of the Capri applicator

**DOI:** 10.1120/jacmp.v15i1.4581

**Published:** 2014-01-06

**Authors:** Aime M. Gloi

**Affiliations:** ^1^ Radiation Onclogy Department St Vincent Hospital Green Bay WI USA

**Keywords:** NTCP, TCP, EUD, quality factors, gynecologic brachytherapy

## Abstract

This study was to assess the Capri applicator for patients with endometrial cancer undergoing high‐radiation dose treatments following external‐beam radiation therapy. The Capri applicator is an inflatable vaginal cylinder with multiple channels. It is used to tailor the dose distribution to an asymmetric vaginal disease, and better spare organs at risk. Five patients with high‐risk endometrial cancer were selected for this study. The patients were treated with a high dose of radiation using the Capri applicator: daily fraction of 7 Gy was prescribed for a total dose of 21 Gy. The treatment plans included radiobiological parameters such as equivalent uniform dose (EUD), normal tissue complication probability (NTCP), and tumor control probability (TCP). Based on the dose‐volume histograms (DVH), we also calculated four quality factors: conformity index (CI), dose homogeneity index (DHI), dose nonuniformity index (DNR), and overdose index (OI). The TCP values range from 82.26% to 95.92%. Very low values of NTCP were observed for the bladder and rectum. The EUDs to organs at risk ranged from 4.65 Gy to 18.22 Gy for the bladder, and from 3.41 Gy from to 6.56 Gy for the rectum. The mean CI was 1.05(SD=0.0008). The mean DNR was 0.10(range0.0−0.295,SD=0.100). The mean OI was 0.019(SD=0.028). The DHIs were in the range of 1.0−0.754(mean0.886,SD=0.116). The use of a multichannel vaginal cylinder may not only help cover extensive vaginal disease, but also reduce the dose to the rectum. This dosimetric analysis shows that rectal doses could be reduced using a multichannel cylinder. However, the dose delivered to the bladder based on EUD calculation may be higher than that obtained with other methods. Each patient must be evaluated independently to determine if a multichannel treatment is appropriate. Clinical followup will show whether this rectal dose sparing translates into a real toxicity improvement.

PACS number: 3.6.96.0

## I. INTRODUCTION

Cancers of the vagina and uterus account for 1%‐2% of all cancers affecting women in the United States. The American Cancer Society estimates that 41,200 new cases of cancer of the uterus were diagnosed in 2006.^(1)^ Several different radiation delivery systems are used to treat gynecological cancers. Intracavity brachytherapy insertion is a key technique in the treatment and management of carcinomas of the uterine cervix. Patients with Stage II endometrial carcinoma typically receive a combination of external beam radiotherapy (EBRT) to treat lymph nodes in the pelvis and high‐dose radiation (HDR) to boost the vaginal cuff. Both methods are essential for inoperable, recurrent, or locally advanced endometrial cancers.

In breast cancer treatment, it is now standard practice to design a cumulative dose distribution that matches the patient's specific anatomy by using multiple dwell positions in single‐channel (MammoSite) and multichannel (Savi, Contura) accelerated partial breast irradiation (APBI).

The treatment of endometrial cancer, on the other hand, does not yet take the patient's anatomy into account. HDR treatment often involves the insertion of rigid cylinders into the vagina. However, air pockets can form between the applicators and the vaginal mucosa, complicating the treatment plan and resulting in a reduced radiation dose.^(2)^ The shapes and positions of the air pockets vary from patient to patient, and even from fraction to fraction. Furthermore, due to the cylindrical geometry of the applicators, it is not possible to tailor the dose distribution in the direction perpendicular to the catheter in cases where the disease is asymmetrical. Finally, the dose to the rectum and bladder cannot be decreased without reducing the overall dose to the target volume.

To overcome these deficiencies of the rigid applicator and to allow for greater flexibility during treatment planning and dose modulation, Varian Medical Systems has devised the Capri applicator. It is similar to the APBI, a mutichannel applicator introduced for transvaginal brachytherapy. The Capri is a lightweight balloon applicator with 13 channels. It is inflated with saline after insertion to adapt to the patient's anatomy, and held in place by a clamp during treatment. The peripheral channels allow the doctor to optimize dwell positions to the tumor location, and to tailor the dose distribution in the vicinity of the rectum and bladder. In this study, for example, in order to limit damage to organs at risk, we did not activate channel #2 (close to the bladder) or channel #5 (close to the rectum).

Admittedly, the small sample size limited the conclusions that could have been drawn from the study model, but we have been comforted by studies done by Peng et al.^(3)^ in which three patients were used with a new disposable multichannel applicator, and Bahadur et al.^(4)^ analyzed data of 10 consecutive patients with cervical cancer treated with external beam radiotherapy to the whole pelvis 45 Gy in 25 fractions followed by high‐dose‐rate (HDR) 21 Gy in 3 fractions. In addition, from 2010‐2012, Smith et al.^(5)^ reported on seven patients with locally advanced low rectal adenocarcinoma (within 12 cm of the anal verge) in a prospective study of neoadjuvant EBT (NCT01226979) at Johns Hopkins Hospital. Finally, Fortunato et al.^(6)^ demonstrate the advantages of endoluminal brachytherapy with high‐dose‐rate (HDR) in primary and recurrent tumor of the bronchial tree using seven patients with primary tumors of the colon, trachea, and lung.

The Capri applicator is CT‐compatible and conforms to the vaginal mucosa due to its variable saline filling capability. In this study, we compare the treatment plans of five patients and assess their efficacy through dose‐volume histograms (DVH) and through the biological parameters of critical structures. Evaluating these parameters will help determine the overall quality of the treatment device.

## II. MATERIALS AND METHODS

Five women with Stage II endometrial carcinoma were selected for this study. Each woman received a combination of external beam radiotherapy (EBRT) to treat regional lymph nodes and HDR brachytherapy to boost the vaginal cuff. The prescribed EBRT dose for all patients was 45 Gy, to be delivered in 1.8 Gy daily fractions. The HDR boost delivered 7 Gy/fraction, for a total of 21 Gy.

**Figure 1:**
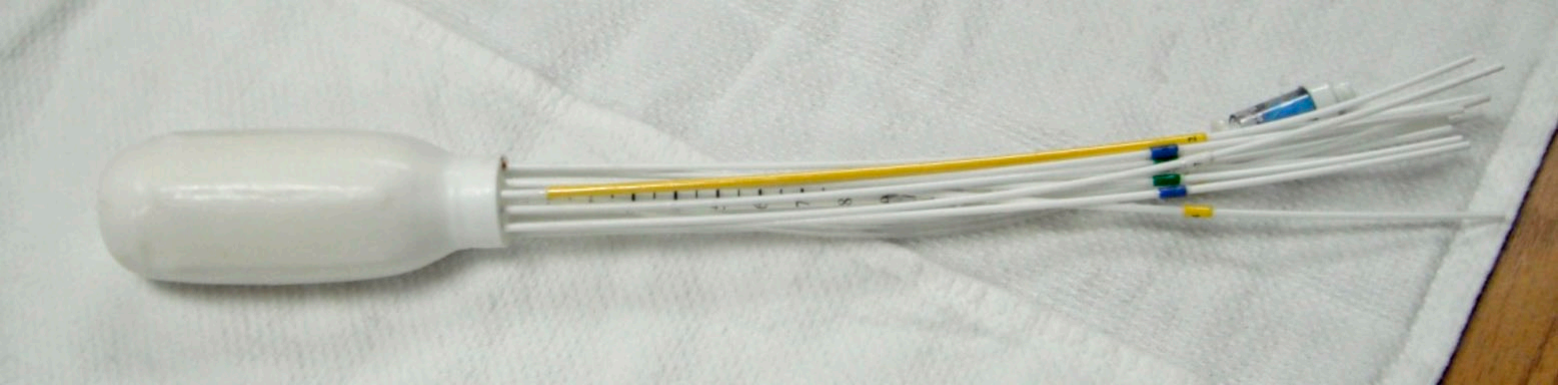
Capri applicator with 13 source lumens for vaginal brachytherapy of endometrial cancer.

The Capri applicator (Varian Medical Systems, Charlottesville, VA) is used to treat cancer of the vagina, vaginal stump, and rectum (Fig. 1). This flexible, inflatable device conforms to the patient's tissues, reducing air gaps and stabilizing the applicator position. Its 13 lumens are arranged in two concentric rings (six lumens per ring, plus one central lumen). This construction allows doctors to shape the dose, providing optimal target coverage while reducing the dose to normal tissues.

In this study, channel #2 (close to the bladder) and channel #5 (close to the rectum) were not used in order to reduce the risk to other organs. A Capri applicator was inserted into the introitus, advanced to the vaginal vault, and then expanded with saline until the applicator was held firmly by the vaginal canal. The applicator was then secured in that position with a clamp. Clinical dose plans were generated from CT images (LightSpeed RT; GE, Milwaukee, WI) using specialized planning software (BrachyVision; Varian Medical Systems, Palo Alto, CA). The primary target volume (PTV_eval) was defined as a 1 cm uniform expansion of the Capri applicator surface, except where this volume encroached on the bladder or rectum. The target volume was recorded in each dose plan. The space occupied by the Capri applicator itself was excluded from PTV_eval. All the patients involved in this study had comparable ages and tumor stages.

### A. Radiobiological modeling

Rectal NTCPs (normal tissue complication probabilities) were estimated using the Lyman‐Kutcher‐Burman (LKB)^(7)^ model for rectal bleeding. The parameters of this model are $\alpha =0.3\;{\text{Gy}}^{-1},\alpha /\beta =3.0\;\text{Gy}$, the volume effect n=0.085, the slope m=0.27, and TD50=97.70Gy. Bladder contracture was assessed with the model parameters α=0.3Gy−1,α/β=3.0Gy,n=0.5,m=0.11, and TD50=80Gy. A computer program (BioSuite; Clatterbridge, UK)^(8)^ was used to perform the EUD calculations. The concept of EUD was first initiated by Andrzej Niemierko in 1997 and was based on the linear quadratic cell survival formalism. The intent was to define the biologically equivalent dose that, if given uniformly, would lead to the same biological effect as the actual nonuniform dose distribution.^(9)^ We used Niemierko's model to evaluate TCP and NTCP based on the individual dose‐volume histograms (DVHs). The probability of controlling a tumor is defined as the probability of having zero surviving clonogenic cells in the tumor at the end of the radiotherapy treatment.^(9)^ In this ideal case, the tumor is unable to produce progeny and is considered to be dead. The TCP if some clonogens remain is calculated from the surviving fraction S using the Poisson equation:

(1) $$TCP=e^{-kS}$$

where *k* is equal to 200. To account for dose heterogeneity, the survival fraction is calculated based on the DVH using the formula:

(2) $$S=\sum_{i}\frac{V_{i}}{V}S(D_{i})$$

where *V* is the total planned treatment volume (PTV_eval) and $V_{i}$ is the subvolume corresponding to dose bin $D_{i}$ in the DVH. This calculation assumes that the density of tumor clonogens is constant throughout the tumor.

The EUD concept is defined as the uniformly distributed dose that leads to the same level of cell killing as the actual, nonuniform dose distribution. Niemierko's model describes the following relationship between EUD and TCP:^10^, ^11^

(3) $$TCP=1/\left[1+{\left(\frac{TCD_{50}}{EUD}\right)}^{\gamma_{50}}\right]$$

where ${\text{TCD}}_{50}$ is the dose required to control 50% of tumors when the tumor is homogeneously irradiated, and $\gamma_{50}$ is a unitless parameter specific to the tumor of interest. Gamma describes the slope of the dose response curve.^(10)^ This model can be used for both tumors and normal tissues. Several quality factors were calculated in this study, as used by various reports in the literature.^12^, ^13^

### B. Conformity index

The conformity index (CI) is the ratio of the volume of tissue receiving at least 95% of the prescribed dose to the total volume in PTV_eval. The CI value should be close to 1.

(4) $$CI=\frac{V_{95}}{V_{PTV\_eval}}$$

### C. Dose homogeneity index

The dose homogeneity index (DHI) is defined as the ratio of the volume that receives a dose in the range of 1.0 to 1.5 times the reference dose to the volume that receives a dose equal to or greater than the reference dose. A higher DHI indicates worse homogeneity.

(5) $$DHI=\frac{V_{100}-V_{150}}{VPTV\_eval}$$

### D. Dose nonuniformity index

The dose nonuniformity index (DNR) is the ratio of the volume that receives a dose equal to or greater than 1.5 times of the reference dose to the volume that receives a dose equal to or greater than the reference dose.

(6) $$DNR=\frac{V_{150}}{V_{100}}$$

### E. overdose volume index

The overdose volume index is the ratio of the target volume that receives a dose equal to or greater than 2.0 times the reference dose to the volume that receives a dose equal to or greater than the reference dose.

(7) $$OI=\frac{V_{200}}{V_{100}}$$

The ideal values of these quality factors are CI=1,DHI=1,OI=0, and DNR=0.

## III. RESULTS

**Figure 2:**
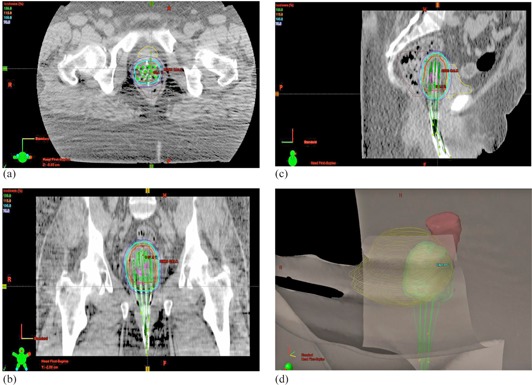
An example of the CAPRI applicator (a 13‐channel applicator) and a dose distribution delivered around the vaginal apex of one patient: (a) axial, (b) sagittal, (c): coronal, and (d) 3D. The 3D image depicts the anatomy and the dose distribution (bladder in yellow, rectum in brown, applicator in green).

**Figure 3:**
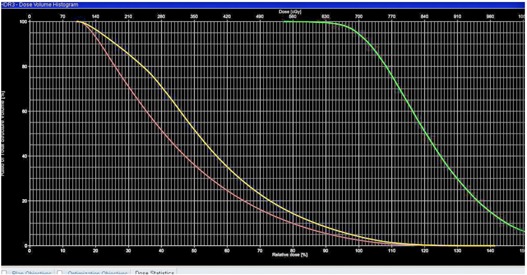
Dose‐volume histograms (DVH) of a patient treated with the Capri applicator. The green curve represents the DVH in PTV_eval, the yellow curve is the bladder, and the brown curve is the rectum.

**Figure 4:**
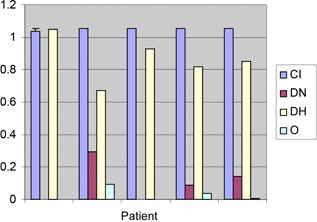
Quality factors for five patients treated with the Capri applicator.

**Figure 5:**
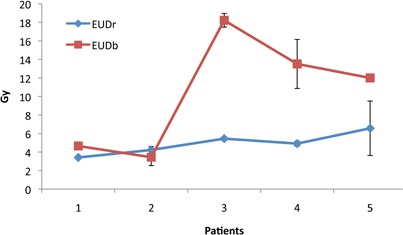
EUDs in the bladder and rectum, for five patients treated with the Capri applicator. The EUD values were calculated through a biological software, BioSuite.

The total dose prescribed to each patient was 21 Gy, delivered in 3 daily fractions of 7 Gy. The same treatment regimen was applied to all five patients in this study. Figures 2(a)‐2(d) illustrate the Capri applicator inside a patient with endometrial cancer. The treatment plans were evaluated both quantitatively via dose‐volume histograms (DVHs; Fig. 3), and qualitatively using the indices and ratios defined in the previous section (Fig. 4). The mean PTV_eval was 33.80 cc (range 16.8‐45.7 cc). The mean EUDs for rectum and bladder were 4.91 Gy and 10.32 Gy, respectively (the raw data are charted in Fig. 5).

In this study, the PTV_eval volume dose was optimized to 95%, while the doses to other organs at risk were minimized. The dose constraints to the other organs at risk are summarized in Table 1. The DVHs drop off steeply for doses beyond the prescribed dose, indicating that the average mean dose within PTV_eval volume is very close to the prescribed dose. Patient #3 required the largest PTV_eval volume and also has the highest EUD to the bladder. This is one example of the wide variation observed in many quality indices, which may be due to different anatomical conditions. Apart from the EUD to the bladder, however, there was no obvious relationship between PTV_eval volume and other dosimetric results.

**Figure 6:**
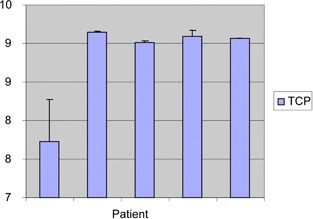
TCPs for five patients treated with the Capri applicator.

The NTCP values obtained in this study are very low for both rectum and bladder, averaging 0.033% and 0.083%, respectively. The average TCP was 93.02% and all TCPs are charted in Fig. 6. In addition, we calculated the four quality indices defined above based on the DVH of each patient. These values are charted in Fig. 4. The mean CI was 1.05(range1.0505−1.0525,SD=0.0008). The mean DNR was 0.1(range0.0−0.295,SD=0.100). The maximum OI was 0.065(mean0.019,SD=0.028). Finally, the DHIs fell in the range 1.0−0.754(mean0.886,SD=0.116).

**Table 1:** Dose constraints for volume optimization. The permitted doses to the bladder and rectum are 80% of the dose prescription

*Organ*	*Volume*	*Dose Constraint (cGy)*	*Priority*
Bladder	0%	<700	100%
Rectum	0%	<700	100%
PTV_Eval	95%	≥550	100%

## IV. DISCUSSION

The radiation dose that can be applied in endometrium cancer with HDR techniques is limited by the doses delivered to the rectum and bladder. This study investigated the radiation dose distribution obtained with a new applicator, the Capri, and quantified its ability to maximize dose in the target and minimize dose to other organs at risk.

In the literature, we could not find any other study permitting a direct comparison of our data, which is derived from Capri applicator treatment plans. Nevertheless, we can cite some studies which measure TCP and NTCP in the pelvic area for HDR therapies. In Dale et al.,^(14)^ 200 patients treated for the whole pelvis had mean NTCPs of 61% and 19.7% for the bladder and rectum, respectively. Huang et al.^(15)^ report average TCPs for cervical cancer ranging from 33% to 100% in 11 distinct patient groups. Using the Capri applicator, on the other hand, this five‐patient study revealed NTCP values close to zero for the rectum and bladder. One reason for these low NTCP values is that all five treatment plans left channels #2 and #5 inactive to minimize risk to nearby organs. This methodology should be taken into consideration when planning the treatment of an endometrial cancer.

Figure 6 reports that the average TCP is 93.02%. TCP is often used as an alternate or supplemental indicator of treatment outcome and higher values are encouraging. The EUD is commonly used in conjunction with TCP to evaluate the overall effectiveness of the treatment and identify potential cold spots in the plan. In this study, the average EUD in the bladder was 10.4 Gy. This value is high compared to the therapeutic dose to the bladder (5.6 Gy, 80% of the dose to PTV_eval). In addition, the EUD in the bladder was highly variable. For both reasons, the bladder EUD is not suitable for controlling the therapeutic dose. On the other hand, the average EUD in the rectum ranges from 3.41 Gy to 6.56 Gy and is highly sensitive to the minimum dose. Therefore, the rectal EUD could be appropriate for managing the therapeutic dose in brachytherapy, where “hot” areas appear in the high‐dose volume. Several DVH data reduction methods have been used to determine TCP and NTCP. In addition, the correct parameters for predicting rectum and bladder outcomes (such as α/β,n,m, and ${\text{TD}}_{50}$ in the LKB model) have some uncertainty. Several studies^16^, ^17^, ^18^ have already focused on these aspects of the problem. For example, the values of α/β used for late rectal toxicity are derived from reports that avoid one or more common sources of uncertainty such as organ motion, variability of dose in each voxel, limited endpoint, and different dose‐volume dependencies. It is worth noting that since TCP and NTCP are calculated from analytical models, any attempts to optimize the dose distribution in relation to these values will have limited success. Another approach could be optimization on the basis of EUD. EUD could be used to assess the therapeutic dose in brachytherapy, where “hot” dose areas are confined in the high‐dose volume. Higher TCP values are often associated with a high, but nonuniform, dose distribution in HDR delivery.

In this study, we also assessed some quality factors in order to assess the treatment plans. For example, CI evaluates the relationship between the isodose distribution and the target volume. A CI of unity is ideal, implying both high PTV_eval coverage and minimal unnecessary irradiation of surrounding tissues. The dosimetric analysis reveals that both CI and DHI were close to one. Note that the mean CI (1.05) and mean DHI (0.88) estimated in our study represent nearly ideal clinical conditions, and similar values should be reproducible by other physicians. This is also true of the low values we observed in NTCP for both bladder and rectum. Taken together, the four quality metrics indicate that using Capri applicator techniques results in a very good dose distribution to the target volume, while delivering significantly smaller region of high doses to the OARs.

The motivation for a multichannel vaginal applicator such as the Capri is based on observed patterns of asymmetric disease geometry and the inability of single‐channel applicators to tailor the dose distribution to the disease site or avoid adjacent OARs. Several alternative techniques of vaginal cuff treatment have been developed in response to this problem, such as interstitial brachytherapy and multichannel HDR. The method of interstitial brachytherapy presents several disadvantages, including the invasiveness of the procedure, the need for an expert, and sometimes hospital stays. It carries the risk of wound infections, abscesses, and fat necrosis, with infection rates approaching 7%. On the other hand, the single‐cylinder HDR often creates air pockets, reducing the radiation dose to the vaginal mucosa.^(2)^

The Capri applicator is noninvasive and employs multiple channels. Its dose distribution can be tailored to minimize the dose to adjacent structures, such as the bladder and rectum. This will result in good PTV_eval coverage. As the Capri applicator is inflatable with saline, it also achieves high conformance with the vaginal mucosa and greatly reduces air pockets.^(19)^ This technique is attractive to patients, as it has a good dose control of the bladder and rectum and can be used to deliver dose at depth laterally (i.e., difficult vaginal cases with gross disease), which in turn will help eliminate the need for further endometrium recurrence. Its main drawback resides in the 3 cm introitus limit.

This study is a preliminary evaluation of the Capri applicator, especially its ability to reduce the dose to adjacent organs. The Capri applicator is also suitable for rectal treatment because it has a variable fill volume.

## V. CONCLUSIONS

The Capri applicator in conjunction with 3D volumetric dose planning techniques allows doctors to create target volumes that conform closely to the patient's anatomy, reducing the dose to adjacent organs while maintaining an acceptable dose to the mucosal surface.
